# Identification of immune hub genes participating in the pathogenesis and progression of Vogt-Koyanagi-Harada disease

**DOI:** 10.3389/fimmu.2022.936707

**Published:** 2022-07-25

**Authors:** Yiqi Wang, Yahan Ju, Jiajing Wang, Na Sun, Zhimin Tang, Huiqin Gao, Ping Gu, Jing Ji

**Affiliations:** ^1^ Department of Ophthalmology, Ninth People’s Hospital, Shanghai Jiao Tong University School of Medicine, Shanghai, China; ^2^ Shanghai Key Laboratory of Orbital Diseases and Ocular Oncology, Shanghai, China; ^3^ Department of Ophthalmology, Xinhua Hospital, Shanghai Jiao Tong University School of Medicine, Shanghai, China

**Keywords:** Vogt-Koyanagi-Harada disease, functional enrichment analyses, protein-protein interaction network analysis, immune hub genes, receiver operating characteristic curves

## Abstract

**Background:**

Vogt-Koyanagi-Harada (VKH) disease is an autoimmune inflammatory disorder characterized by bilateral granulomatous uveitis. The objective of this study was to identify immune hub genes involved in the pathogenesis and progression of VKH disease.

**Methods:**

High throughput sequencing data were downloaded from the Gene Expression Omnibus (GEO) and an immune dataset was downloaded from ImmPort. Immune differentially expressed genes (DEGs) were obtained from their intersection in the GEO and ImmPort datasets. Immune hub genes for VKH disease were selected through differential expression analyses, including Gene Ontology (GO), Kyoto Encyclopedia of Genes and Genomes (KEGG), Disease Ontology (DO), protein-protein interaction (PPI) network, and clustering analyses. Confidence in the immune hub genes was subsequently validated using box plots and receiver operating characteristic (ROC) curves.

**Results:**

A total of 254 DEGs were screened and after the intersection with ImmPort, 20 genes were obtained as immune DEGs. Functional enrichment analysis indicated that the key genes were mainly involved in several types of immune pathways (such as the lymphocyte mediated and leukocyte mediated immune responses, natural killer cell mediated cytotoxicity, and antigen binding) and immunodeficiency diseases. Following PPI network analysis, the top seven genes in cluster 1 were selected as potential immune hub genes in VKH. After evaluating the accuracy of the hub genes, one gene (GNLY) was excluded because its expression level was statistically similar in VKH patients and healthy controls. Finally, six immune hub genes, namely KLRC2, KLRC3 SH2D1B, GZMB, KIR2DL3, and KIR3DL2 were identified as playing important roles in the occurrence and development of VKH disease.

**Conclusion:**

Six immune hub genes (KLRC2, KLRC3 SH2D1B, GZMB, KIR2DL3, and KIR3DL2) identified by our bioinformatics analyses may provide new diagnostic and therapeutic targets for VKH disease.

## Introduction

Vogt-Koyanagi-Harada (VKH) disease is an autoimmune inflammatory disorder with both intraocular and extraocular manifestations. Heavily pigmented races, such as Asians (1–3), Latin Americans (4, 5), and Middle Easterners (6–8), have high incidence rates of VKH disease, women are more susceptible than men (9, 10) and 20-to-50-year-olds are primarily affected (11). The disease progresses through prodromal, acute uveitic, chronic convalescent and chronic recurrent phases (12) and patients characteristically present with bilateral granulomatous uveitis. Retinal detachments, disk edema, vitritis and eventually sunset glow fundus are typical intraocular findings in VKH disease, accompanied by a series of systemic symptoms, such as headache, tinnitus, poliosis, vitiligo, and meningismus (13). The etiology and pathogenesis of the disease have not been fully clarified. Although melanocytes have been widely acknowledged as targets of autoimmune responses in VKH disease (14, 15), microbial infection (16), gene susceptibility (17), immune-related cells and pathways also have vital roles in its pathogenesis. For example, CD4+ T cells, Th1 cells, Th17 cells, and a series of cytokines have been found to be associated with the disease (14, 18–22). However, the underlying molecular mechanisms of the pathogenesis of VKH disease remain to be elucidated. Therefore, further research is essential to better understand its occurrence and progression.

Since the ocular tissue samples of patients with VKH disease (such as aqueous humor and choroid) are difficult to obtain, basic research on the pathogenesis of this disease is challenging, limiting the scope to further understand the mechanisms of immune-related cells and pathways involved in its progression. The rapid development of high throughput sequencing technology has facilitated the availability of key genes for the identification of diagnostic and therapeutic biomarkers of VKH disease. Since blood samples are easier to obtain than ocular tissues, evaluating gene expression in the peripheral blood of VKH patients may help enhance comprehension of the pathogenesis of VKH disease. To date, no bioinformatics analysis has focused on the mechanism of immune genes in VKH disease. Thus, we analyzed VKH disease-related datasets to screen differentially expressed genes (DEGs) in peripheral blood from VKH patients and healthy controls and then intersected these DEGs with an immune dataset to obtain immune DEGs. Subsequently, functional enrichment analyses of immune DEGs were conducted. Protein-protein interaction (PPI) network and clustering analyses were conducted to identify potential immune hub genes, and their value in clinical diagnosis of VKH disease was predicted using receiver operating characteristic (ROC) curves.

## Materials and methods

### High throughput sequencing data

Clinical data from patients with VKH disease and healthy controls were obtained from the Gene Expression Omnibus (GEO, https://www.ncbi.nlm.nih.gov/geo/). The dataset GSE166663 was downloaded, including a total of 14 samples, among which seven were from VKH patients and seven from healthy controls. The consent of patients and the approval of ethics committee were unnecessary because the data were downloaded from public databases.

### Differential expression analysis

The “limma” and “pheatmap” packages of R software (version 4.1.3) ( https://www.r-project.org/) were used to perform differential expression analysis and to draw a heatmap. The expression profiles of VKH patients and healthy controls were compared to identify the DEGs. A t-test was used to determine P-values in DEG analysis.

Genes retained were selected using the criteria of P-value <0.05 and |log2(Fold Change)|>1. A dataset including 1793 genes from the immune database (ImmPort, https://www.immport.org) was also obtained and intersected with GSE166663 to identify immune DEGs. The “VennDiagram” package of R software (version 4.1.3) was employed to generate a Venn diagram.

### Functional enrichment analyses

The “clusterProfiler”, “org.Hs.eg.db”, “enrichplot”, “ggplot2”, “GSEABase”, and “DOSE” packages of R software (version 4.1.3) were used to obtain the Gene Ontology (GO) functions, Kyoto Encyclopedia of Genes and Genomes (KEGG) enrichment, and to conduct Disease Ontology (DO) analyses of immune DEGs and visualize the obtained data. P values <0.05 were considered statistically significant.

### Protein-protein interaction network

The STRING database (https://cn.string-db.org/) was utilized to predict PPIs with the protein species set to “HomoSapiens” and the lowest interaction threshold set to “low confidence” (0.15). The PPI network was then visualized using Cytoscape software (https://cytoscape.org/). Further clustering analysis was conducted using molecular complex detection (MCODE), an application of Cytoscape. Genes in the key sub-network were selected as potential immune hub genes.

### Validation of hub genes

Box plots were drawn using the “ggpubr” package of R software (version 4.1.3) and showed the potential immune hub gene expression levels in VKH patients and healthy controls. ROC curves were drawn using the “pROC” package of R software (version 4.1.3) to assess the levels of potential immune hub genes in VKH disease. Immune hub genes were selected using the criteria of P-value <0.05 and area under curve (AUC) value >0.8.

## Results

### Identification of immune DEGs

**Figure 1:**
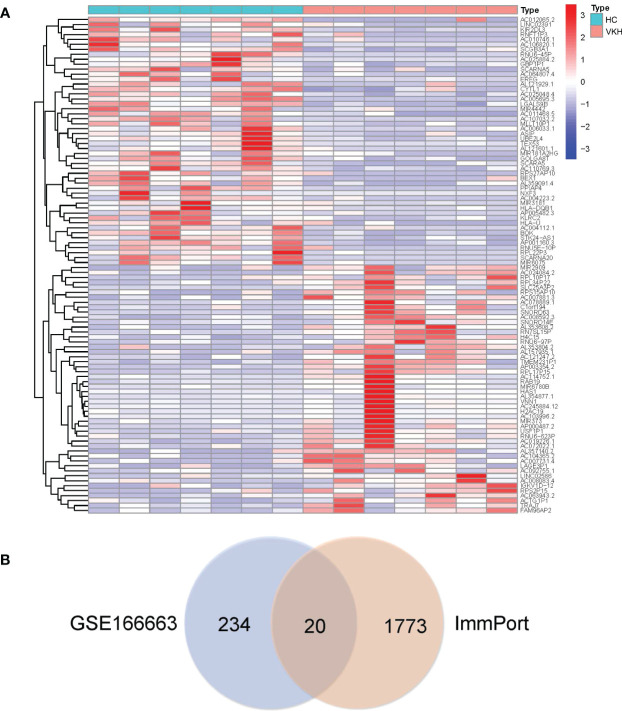
In total, 254 differentially expressed genes (DEGs) were identified in Vogt-Koyanagi-Harada (VKH) patients and healthy controls (HC) and the top 50 DEGs are shown in **(A)** the heatmap, with significantly up-regulated genes marked in red and significantly down-regulated genes marked in blue. **(B)** Venn diagram of immune DEGs. Clinical dataset of VKH disease (GSE166663) and immune dataset (ImmPort) were intersected to identify immune DEGs.

A high throughput sequencing dataset, GSE166663, was downloaded from GEO and we compared VKH patients with healthy controls to obtain DEGs. The top 50 DEGs identified from the high throughput sequencing dataset are shown in the heatmap (**Figure 1A**). Intersection analysis identified 13 downregulated genes and seven upregulated genes (**Table 1**). The Venn diagram of immune DEGs is shown in **Figure 1B**.

**Table 1:** Immune differentially expressed genes of Vogt-Koyanagi-Harada disease.

Gene symbol	log_2_FC	P-value	Entrez ID
HLA-DQB1	-2.10	0.0262	3119
KIR2DL3	-1.87	0.0006	3804
KIR3DL2	-1.19	0.0070	3812
KLRC2	-2.04	0.0400	3822
KLRC3	-1.04	0.0379	3823
SFTPD	-1.57	0.0111	6441
GNLY	-1.11	0.0041	10578
PLXNA4	-1.02	0.0262	91584
EREG	-3.09	0.0151	2069
SCGB3A1	-2.21	0.0048	92304
SH2D1B	-1.78	0.0006	117157
GZMB	-1.56	0.0006	3002
TRDJ1	-1.55	0.0070	28522
HSPA1A	1.14	0.0379	3303
SLPI	1.54	0.0262	6590
IGHV2-26	1.75	0.0252	28455
IGHV3-15	1.57	0.0007	28448
IGKV1D-12	1.93	0.0200	28903
IGLV8-61	1.37	0.0262	28774
TRAJ7	2.29	0.0007	28522

### Functional enrichment analysis of immune DEGs

**Figure 2:**
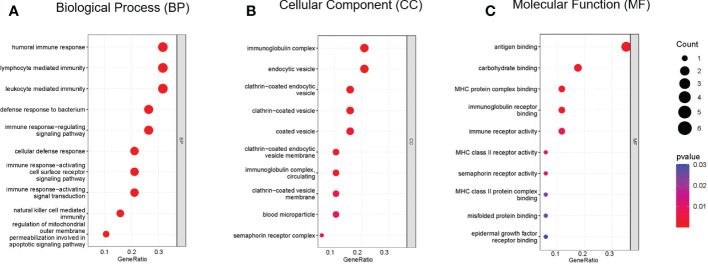
Bubble plot of gene ontology (GO) functional analysis included the respective top 10 terms in **(A)** biological process, **(B)** cellular component, and **(C)** molecular function, indicating that the immune differentially expressed genes (DEGs) were mainly involved in humoral immune response, lymphocyte mediated immunity, leukocyte mediated immunity, and antigen binding (*P*<0.05).

**Figure 3:**
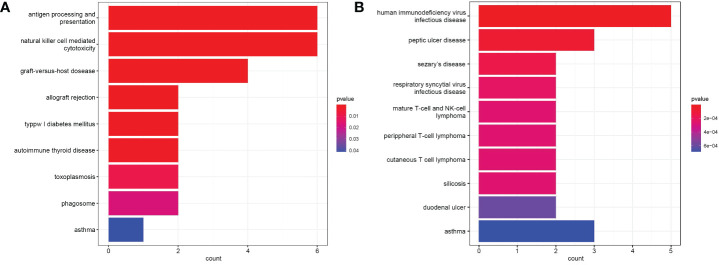
**(A)** Bar plot of results from Kyoto Encyclopedia of Genes and Genomes (KEGG) functional analysis indicating that antigen processing and presentation, natural killer cell mediated cytotoxicity, and graft-versus-host disease were most significantly activated in the gene sets (*P*<0.05). **(B)** Bar plot of disease ontology (DO) functional analysis showed the top 10 diseases, with the immune differentially expressed genes (DEGs) primarily participating in the pathogenesis of human immunodeficiency virus infectious disease (*P*<0.05).

Functional enrichment analysis of immune DEGs was performed using R software (version 4.1.3). The results of GO analysis revealed that the significantly enriched genes were mainly involved in humoral immune response, lymphocyte mediated and leukocyte mediated immunity, and antigen binding (**Figure 2**). KEGG analysis indicated that antigen processing and presentation, natural killer cell mediated cytotoxicity, and graft-versus-host disease were significantly enriched in the gene sets (**Figure 3A**). Finally, DO analysis showed that the immune DEGs primarily participated in the progression of human immunodeficiency virus infectious disease (**Figure 3B**).

### Protein-protein interaction network analysis of immune DEGs

**Figure 4:**
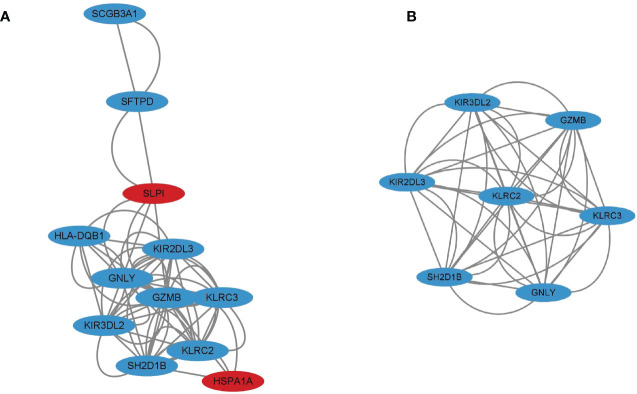
**(A)** Protein-protein interaction (PPI) network for 12 immune differentially expressed genes (DEGs) with the upregulated genes marked in red and the downregulated genes marked in blue. **(B)** Cluster 1 consisting of seven genes was constructed using MCODE.

PPI network analysis was performed using the STRING online database and Cytoscape software. After removing eight genes which were not related to other molecules, the PPI network contained a total of 12 nodes and 31 edges. In **Figure 4A**, nodes represent genes and edges represent interactions between genes, with the upregulated genes marked in red and downregulated genes marked in blue. Clustering analysis established one key module (**Figure 4B**) and seven key genes (KIR2DL3, KIR3DL2, SH2D1B, KLRC3, KLRC2, GNLY, and GZMB) identified using MCODE were selected as potential hub genes for VKH disease.

### Identification of immune hub gene expression levels and diagnostic values

**Figure 5:**
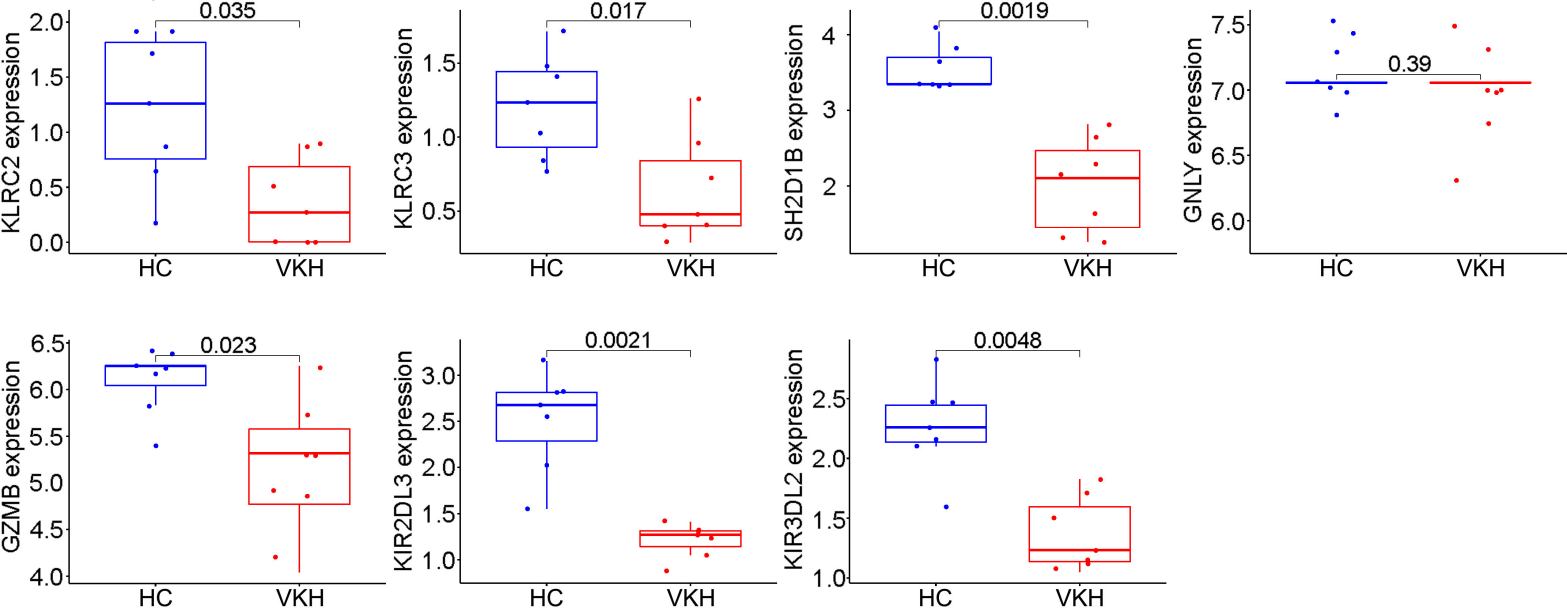
Validation of immune potential hub genes in the gene expression level. The expressions of KLRC2, KLRC3, SH2D1B, GZMB, KIR2DL3, and KIR3DL2 were significantly lower in Vogt-Koyanagi-Harada patients (VKH) than in healthy controls (HC). The expression level of GNLY was not statistically significantly different between the two groups.

**Figure 6:**
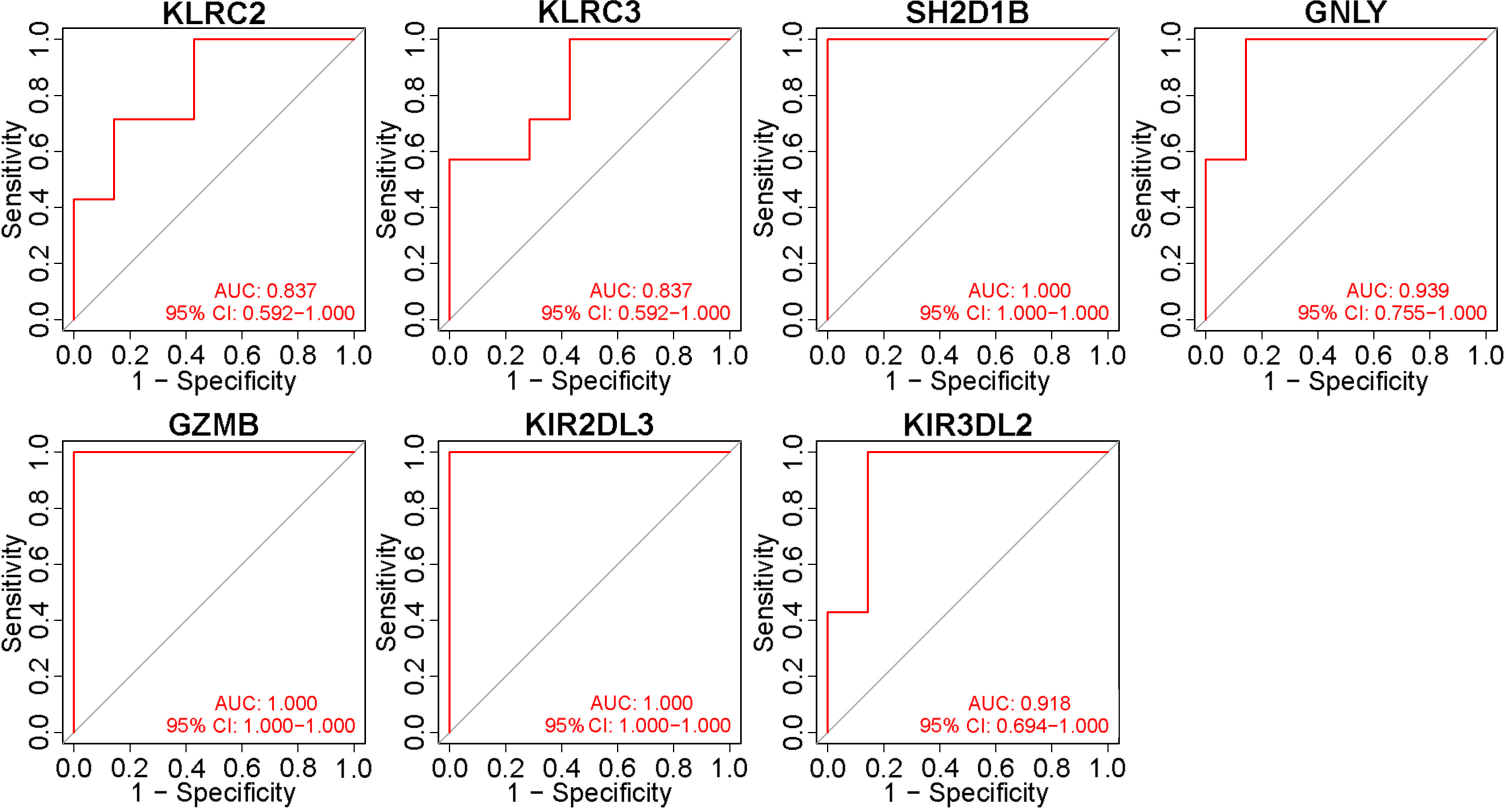
Validation of the diagnostic value of immune potential hub genes. Receiver operating characteristic values and area under the curve (AUC) statistics indicated that all seven potential hub genes had diagnostic value for Vogt-Koyanagi-Harada disease.

To validate these potential immune hub genes, both the box plots and the ROC curves with AUC calculated were made with R software (version 4.1.3). Box plots were used to validate the expression levels of the seven immune potential hub genes (**Figure 5**). Significantly lower expression levels of KLRC2 (P=0.035), KLRC3 (P=0.017), SH2D1B (P=0.0019), GZMB (P=0.023), KIR2DL3 (P=0.0021), and KIR3DL2 (P=0.0048) were found in VKH patients than in healthy controls. However, the expression level of GNLY (P>0.05) showed no statistically significant difference between the two groups. Thus, GNLY could not be considered as an immune hub gene in VKH disease. ROC curves were used to assess the sensitivity and specificity of the key genes in diagnosing VKH disease (**Figure 6**). The AUC values >0.8 in all seven potential hub genes indicated their diagnostic value for VKH diseases. After excluding GNLY as explained above, KLRC2, KLRC3 SH2D1B, GZMB, KIR2DL3, and KIR3DL2 were validated as six immune hub genes in both expression levels and diagnostic values.

## Discussion

In the present study, immune-related key genes involved in VKH disease were identified and the role of immune mechanisms in VKH disease was furtherly explored. Moreover, six immune hub genes were identified and verified as having diagnostic value in VKH disease. The results of both GO and KEGG enrichment analyses indicated that the gene sets were primarily enriched in several types of immune pathways, including lymphocyte mediated and leukocyte mediated immune responses, natural killer cell mediated cytotoxicity, and antigen binding, which are closely related to the pathogenesis of VKH disease. In addition, DO analysis suggested that the immune DEGs were mainly involved in human immunodeficiency virus infectious disease. As VKH disease is an autoimmune inflammatory disorder with virus infection playing an important role in its pathogenesis (12, 23), the results of the present DO analysis were consistent with those of previous studies. Since monocytes were thought to be significantly involved in the development of autoimmune diseases, peripheral blood mononuclear cells (PBMCs) have gradually become a breakthrough in studying the pathogenesis of VKH disease. Single-cell RNA sequencing (scRNA-seq), the latest advanced technology, has been used in a recent study, identifying six subpopulations of human blood monocytes, among which the proinflammatory monocyte subpopulation is a promising therapeutic target for treating VKH disease (24). Another research purified the CD4+ T cells from PBMCs and extracted total RNA of CD4+ T cells. RNA-seq was conducted, revealing that circular RNAs (cicRNAs) may have an important immunomodulatory function in the development of VKH disease (25). As the existing researches have revealed the crucial role of immune cells and molecules in the development of VKH disease, our research group innovatively intersected the screened DEGs between VKH patients and healthy controls with the immune dataset and finally found out six immune hub genes in VKH disease, furtherly providing a new insight into the pathogenesis and treatment of this disease.

It has been revealed in previous studies that autoimmune response against melanin in multiple organs may cause the clinical manifestations of VKH disease and that cell mediated immune responses play an important role in its occurrence and progression (26–28). Among all the immune-related cells, T cells have proven crucial in the development of VKH diseases. Since the experimental autoimmune uveitis model of VKH became widely accepted in the 1990s, several animal experiments have indicated that with leukocyte infiltration of the retina, CD4+ T cells, Th1 cells and Th17 cells may be activated to trigger autoimmune responses and ocular inflammation (29, 30). Since it is difficult to obtain aqueous humor or choroid samples from VKH patients, studies of peripheral blood lymphocytes have been widely conducted to identify the immune responses involved in human VKH disease. Those studies have shown that CD4+CD25high Treg cells, which may suppress the proliferation of CD4+CD25- T cells, are deficient in VKH patients (31), clarifying how CD4+ T cells cause autoimmune responses in affected human eyes. In addition, Th1 cells (14, 19, 21), Th 17 cells (18, 32), and their related cytokines (such as IL-6, IL-12, IL-17, IL-23, and IFN-γ) were found to be involved in the pathogenesis of human VKH disease. The finding that uveal pigment is an antigen in the development of VKH disease (33) suggested that antigen binding plays an important role in its pathogenesis. It has been widely accepted that the combination of CD4+ T cells and melanocyte-related proteins plays an important role in the pathogenesis of VKH disease (34). In addition, Sugita et al. found that MART-1 is a vital antigen in HLA-A2+ VKH patients (15). Tyrosinase family proteins, especially TRP1 and TPR2, have also been found to play a part in the progression of VKH disease (35). These previous studies provide context for the results of our functional enrichment analyses and indicate that the immune DEGs are pivotal in the pathogenesis and development of VKH disease.

Among the six immune hub genes identified in the present analysis, KIR2DL3 and KIR3DL2 have been found to be related to VKH disease. KIR2DL3 and KIR3DL2, members of inhibitory killer immunoglobulin-like receptors (KIR), may prevent the activation of natural killer cells and T cells (36). A number of KIR genes and human leukocyte antigen (HLA) genes (ligands for KIRs) have previously been used to analyze the gene susceptibility of autoimmune diseases (37). Studies have shown that some inhibitory KIR-HLA combinations are lower in VKH patients than in healthy controls, consistent with our bioinformatics analysis results. In addition to inhibitory KIRs, activating KIRs also play an important part in the pathogenesis in VKH disease, with higher levels of activating KIR-HLA bindings in VKH patients than in healthy controls (38–40). Other immune hub genes are also key to the human immune system. KLRC2 and KLRC3 are both members of killer cell lectin like receptor C (KLRC), a gene family located within the natural killer complex, which can regulate specific humoral and cell-mediated immunity. Although there has been no research to date on the role of KLRCs in the pathogenesis of VKH disease, they have been found to be involved in the development of other autoimmune diseases and tumors. For example, Fatma et al. found that KLRC2+ CD4+ T cells target oligodendrocytes in multiple sclerosis (41). Moreover, the study conducted by Mathilde et al. found that KLRC3 overexpressed in glioblastoma undifferentiated cells and furtherly revealed that the gene expression of KLRC3 was related to glioblastoma aggressiveness (42). SH2 domain containing 1 B (SH2D1B) regulates signal transduction through receptors expressed on the surface of antigen-presenting cells. It is mainly expressed in innate immune cells and the expression of SH2D1B is associated with antigen presentation in human cells. Yasser et al. exposed human cells to SH2D1B-overexpression vaccines, and found that SH2D1B could improve antigen presentation in innate immune cells (43). Granzyme B (GZMB) can encode proteins, mainly including the granzyme subfamily of proteins and peptidase S1 family of serine proteases, process cytokines, and degrade extracellular matrix proteins, thus playing an important role in chronic inflammation. Similar to KLRCs, there have been studies showing its role in autoimmune diseases. For example, overexpression of CD4+ GZMB+ CTL cells were found in Sjögren’s syndrome (44). GZMB was also proved important in the pathogenesis of inflammatory skin diseases due to GZMB-mediated proteolysis involved in processes such as tissue remodeling and autoantigen generations (45). All the six immune hub genes identified in the present study are relevant to autoimmune responses, and further research is needed to better understand their involvement in VKH disease.

## Conclusion

All in all, we obtained 20 immune DEGs (13 downregulated genes and seven upregulated genes) in VKH disease and finally screened out six immune hub genes (KLRC2, KLRC3 SH2D1B, GZMB, KIR2DL3, and KIR3DL2) associated with VKH disease, some of which have not been mentioned in the present researches of VKH disease until now. As far as we know, this is the first research to find out immune hub genes in the pathogenesis of VKH disease. Further analyses validating the expression levels and diagnostic levels of these hub genes are of great significance to provide new diagnostic and therapeutic targets of VKH disease in future works. However, it should be noted that this study only includes bioinformatic analyses, thus, further experimental validations are necessary.

## Data Availability Statement

The datasets presented in this study can be found in online repositories. The names of the repository/repositories and accession number(s) can be found in the article/supplementary material.

## Ethics Statement

Ethical review and approval were not required for the study on human participants in accordance with the local legislation and institutional requirements. Written informed consent for participation was not required for this study in accordance with the national legislation and the institutional requirements.

## Author Contributions

JJ, PG, and YW designed the experiments. YW performed the experiments. YW, YJ, JW, NS, ZT, and HG wrote the manuscript and analyzed the data. All authors contributed to the article and approved the submitted version.

## Funding

This study was supported by the medical-engineering cross fund of Shanghai Jiao Tong University (No. YG2021ZD13), The Science and Technology Commission of Shanghai (No.20DZ2270800).

## Acknowledgments

We sincerely thank all the researches for the relevant studies and the publicly available datasets.

## Conflict of Interest

The authors declare that the research was conducted in the absence of any commercial or financial relationships that could be construed as a potential conflict of interest.

## Publisher’s Note

All claims expressed in this article are solely those of the authors and do not necessarily represent those of their affiliated organizations, or those of the publisher, the editors and the reviewers. Any product that may be evaluated in this article, or claim that may be made by its manufacturer, is not guaranteed or endorsed by the publisher.
